# Method of Ga removal from a specimen on a microelectromechanical system-based chip for in-situ transmission electron microscopy

**DOI:** 10.1186/s42649-020-00043-6

**Published:** 2020-10-14

**Authors:** Yena Kwon, Byeong-Seon An, Yeon-Ju Shin, Cheol-Woong Yang

**Affiliations:** 1 School of Advanced Materials Science and Engineering, Sungkyunkwan University, Suwon, 16419 Republic of Korea; 2 Cooperative Center for Research Facilities, Sungkyunkwan University, Suwon, 16419 Republic of Korea

**Keywords:** Focused ion beam, Ex-situ lift-out system, Ga residue, MEMS-based chip, Ar^+^ ion milling

## Abstract

In-situ transmission electron microscopy (TEM) holders that employ a chip-type specimen stage have been widely utilized in recent years. The specimen on the microelectromechanical system (MEMS)-based chip is commonly prepared by focused ion beam (FIB) milling and ex-situ lift-out (EXLO). However, the FIB-milled thin-foil specimens are inevitably contaminated with Ga^+^ ions. When these specimens are heated for real time observation, the Ga^+^ ions influence the reaction or aggregate in the protection layer. An effective method of removing the Ga residue by Ar^+^ ion milling within FIB system was explored in this study. However, the Ga residue remained in the thin-foil specimen that was extracted by EXLO from the trench after the conduct of Ar^+^ ion milling. To address this drawback, the thin-foil specimen was attached to an FIB lift-out grid, subjected to Ar^+^ ion milling, and subsequently transferred to an MEMS-based chip by EXLO. The removal of the Ga residue was confirmed by energy dispersive spectroscopy.

## Introduction

In-situ transmission electron microscopy (TEM), an analytical technique that allows the real-time observation of the microstructural evolution induced by external stimuli such as heating, electrical biasing, and mechanical deformation, is an important current research topic. There has been a recent increase in the use of microelectromechanical system (MEMS)-based chips for in-situ TEM experiments; consequently, the preparation of the specimens for these experiments has become challenging. The fabrication of thin lamellae via a focused ion beam (FIB) (Duchamp et al. 2014; Mele et al. 2016; Vijayan et al. 2017) is one of the most widely used specimen preparation methods for in-situ TEM experiments. This method facilitates the preparation of specimens of uniform thickness within the FIB system; however, the supporting film in the chip may be torn or the surface of specimen may be damaged due to FIB milling. The lamellae are positioned on the MEMS-based chips by lift-out techniques within the FIB system i.e., in-situ lift-out. However, such techniques require multiple complex manipulations and a special inclined stage to minimize the ion flux on the chip during FIB milling (Duchamp et al. 2014; Vijayan et al. 2017; Pivak et al. 2017).

The aforementioned limitations can be overcome by preparing the MEMS-based chip specimens by ex-situ lift-out (EXLO) of the FIB-milled lamellae. EXLO was the first lift-out technique to be implemented for FIB-milled specimens and has been applied to various material systems (Heringer et al. 1996; Giannuzzi et al. 1997). It is a simple, fast technique that takes less than 5 min to manipulate a specimen for analysis (Giannuzzi et al. 2015) and facilitates the precise positioning of the FIB-milled specimens during the transfer onto the MEMS-based chips. However, a major drawback of the technique is the accumulation of residual Ga in the specimen during FIB milling. Several methods have been explored to remove the residual Ga from the specimen; for e.g., the final thinning of the specimen has been conducted using a low-energy ion beam (less than 3 keV) within the FIB system. The removal of the Ga^+^ ions from the specimen outside the FIB system has been explored using plasma cleaner with a bias (Ko et al. 2007), NanoMill (Fischione, USA) (Unoci et al. 2010) and PicoMill (Fischione, USA) (Campin et al. 2018). However, it is difficult to apply these methods to FIB-milled thin specimens in a trench that is subjected to EXLO. In addition, it is difficult to simultaneously remove Ga^+^ ions from both sides of the specimen during the transfer onto the MEMS-based chip. This study demonstrated a comparison of the thin-foil specimens that were fabricated by FIB milling/EXLO with those fabricated by conventional mechanical polishing/ion milling and graphene encapsulation; thus, the problems induced by the accumulation of Ga^+^ ions were observed. Furthermore, a modified method of performing Ar^+^ ion milling within the FIB system was proposed to remove the accumulated Ga^+^ ions.

## Materials and methods

### Deposition

Si (100) wafers were cleaned thrice with ethanol and acetone to remove the organic contaminants from their surface. Subsequently, the Si wafers were immersed in a dilute hydrofluoric acid (HF, 50%) solution (H_2_O:HF = 10:1) to remove the native oxide layer. Thereafter, a 30-nm-thick Ni and 30-nm-thick TaN layer were sequentially deposited on one Si wafer while a 30-nm-thick Er and 30-nm-thick TaN layer were sequentially deposited on the other Si wafer by DC magnetron sputtering.

### Fabrication of thin-foil specimen via mechanical polishing and ion milling

To prepare thin-foil TEM specimens by mechanical polishing and ion milling, the metal-deposited Si wafer was cut in half and the metal-deposited sides were bonded to each other using G-1 epoxy (Gatan Inc., Pleasanton, California, USA). This sandwich wafer was subsequently cut into small pieces (1 × 1.5 mm) using a low-speed diamond saw for further thinning. The cut specimens were mechanical polished using SiC paper, diamond suspensions (6 μm, 3 μm, and 1 μm,), and colloidal silica (0.05 μm), in sequence. The flat polished specimen was attached to a Mo TEM aperture grid with 1.5-mm hole. The specimen was further thinned by Ar^+^ ion milling (PIPS, Gatan Inc., Pleasanton, California, USA) in the dual-beam modulation mode at an accelerating voltage and milling angle of 4 kV and 2° ~ 4° (for both the top/bottom guns), respectively.

### Fabrication of thin-foil specimen on MEMS-based chip via FIB and EXLO technique

**Fig. 1:**
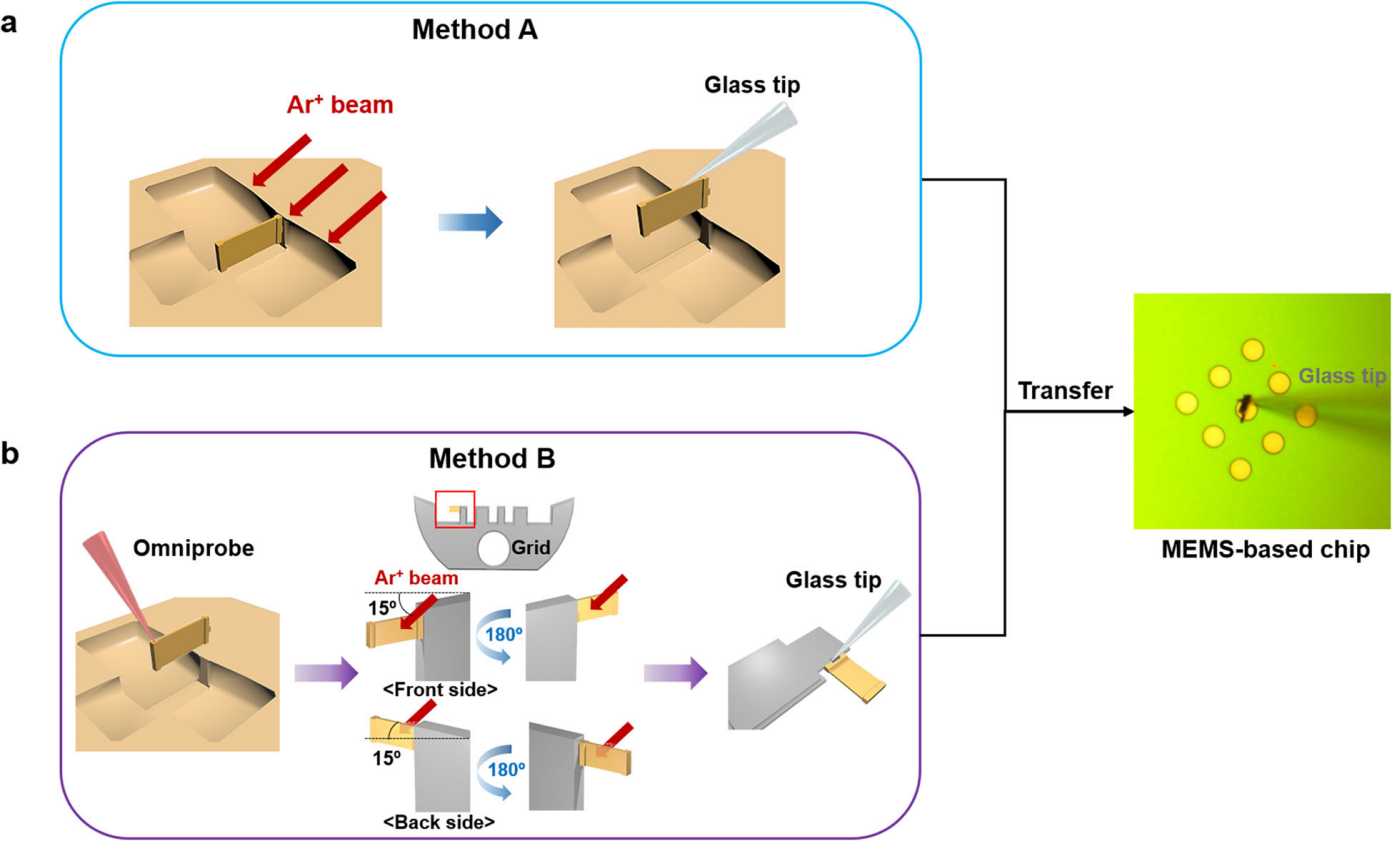
Schematic of the two different methods of Ar^+^ ion milling to remove Ga^+^ ions. **a** Performing Ar^+^ ion milling the lamella while leaving it inside the trench, **b** Performing Ar^+^ ion milling after taking the lamella out of the trench and attacing it to the lift-out grid

The following experiments were conducted in a triple-beam FIB system (NX2000, Hitachi Ltd., Tokyo, Japan). Initially, a protection layer was formed on the metal-deposited Si specimen by sequentially performing electron-beam-induced deposition and ion-beam-induced deposition (IBID) in the FIB system. Subsequently, a high beam current was utilized to create a deep trench, and the specimen was milled using successively lower beam currents as the region of interest was approached. Ar^+^ ion milling was performing in two ways to remove the Ga residue (Fig. 1). The first method was the conventional triple beam FIB milling/EXLO, where the region of interest (thickness < 100 nm) in the trench was milled into the thin-foil specimen. Subsequently, Ar^+^ ion milling was performed, and the thin-foil specimen was lifted and transferred onto an MEMS-based chip by EXLO (Fig. 1(a)). The second method was the proposed modified method, where the specimen was attached to a TEM grid, subjected to Ar^+^ ion milling, and transferred onto an MEMS-based chip by EXLO (Fig. 1(b)). The specimen was milled to a thickness of ~ 1 μm in a trench and attached to an FIB lift-out TEM grid (Nanomesh, Hitachi Ltd., Tokyo, Japan) by IBID of carbon at 30 keV and 80 pA with a dwell time of 0.1 s. Subsequently, the region of interest was etched using a high-energy Ga^+^ ion beam at 30 keV and 1.5 nA, and thinned using a low-energy Ga^+^ ion beam at 5–10 keV and 40 pA; thus, the final thickness of the region of interest was less than 80 nm. Finally, Ar^+^ ion milling was performed at 1 keV and 19 nA in four directions by rotating the specimen by approximately 15° from the initial position. These directions were the front side, back side, front side rotated by 180°, and back side rotated by 180°. The milling was performed for ~ 2–4 min in each direction; furthermore, the final milling was performed in the direction of the front side to prevent the redeposition by the grid. Thereafter, the specimen was detached from the grid by EXLO and transferred onto to the desired area on the MEMS-based chip.

### Fabrication of graphene encapsulated thin-foil specimen

The graphene-encapsulated specimens were fabricated by mechanical exfoilation. The first step of the process included the preparation of a 300-nm-thick SiO_2_/Si wafer and a polymethylmethacrylate (PMMA)/lift-off resist (LOR)/SiO_2_/Si wafer. Subsequently, graphene was separated from highly oriented pyrolytic graphite and transferred onto both wafers. An FIB-milled specimen was transferred onto the prepared graphene/SiO_2_/Si wafer by EXLO. Thereafter, the LOR layer was removed from the graphene/PMMA/LOR/SiO_2_/Si wafer using a KOH solution and deposited on the specimen/graphene/SiO_2_/Si wafer.

### Characterization

The microstructure and elemental composition of the thin-foil specimens were evaluated by TEM (JEM-ARM 200F, JEOL Ltd., Tokyo, Japan) that was performed in conjunction with energy-dispersive X-ray spectroscopy (EDS; INCAEnergy TEM, Oxford Instruments, Oxfordshire, UK) and electron energy loss spectroscopy (EELS; 965 GIF Quantum ER, Gatan Ltd., Pleasanton, California, USA). The in-situ heating experiments were conducted using Fusion Thermal E-chips (E-FHDC, Protochips Inc., Morrisville, North Carolina, USA) and a TEM holder (Fusion 500™, Protochips Inc., Morrisville, North Carolina, USA).

## Results and discussion

**Fig. 2:**
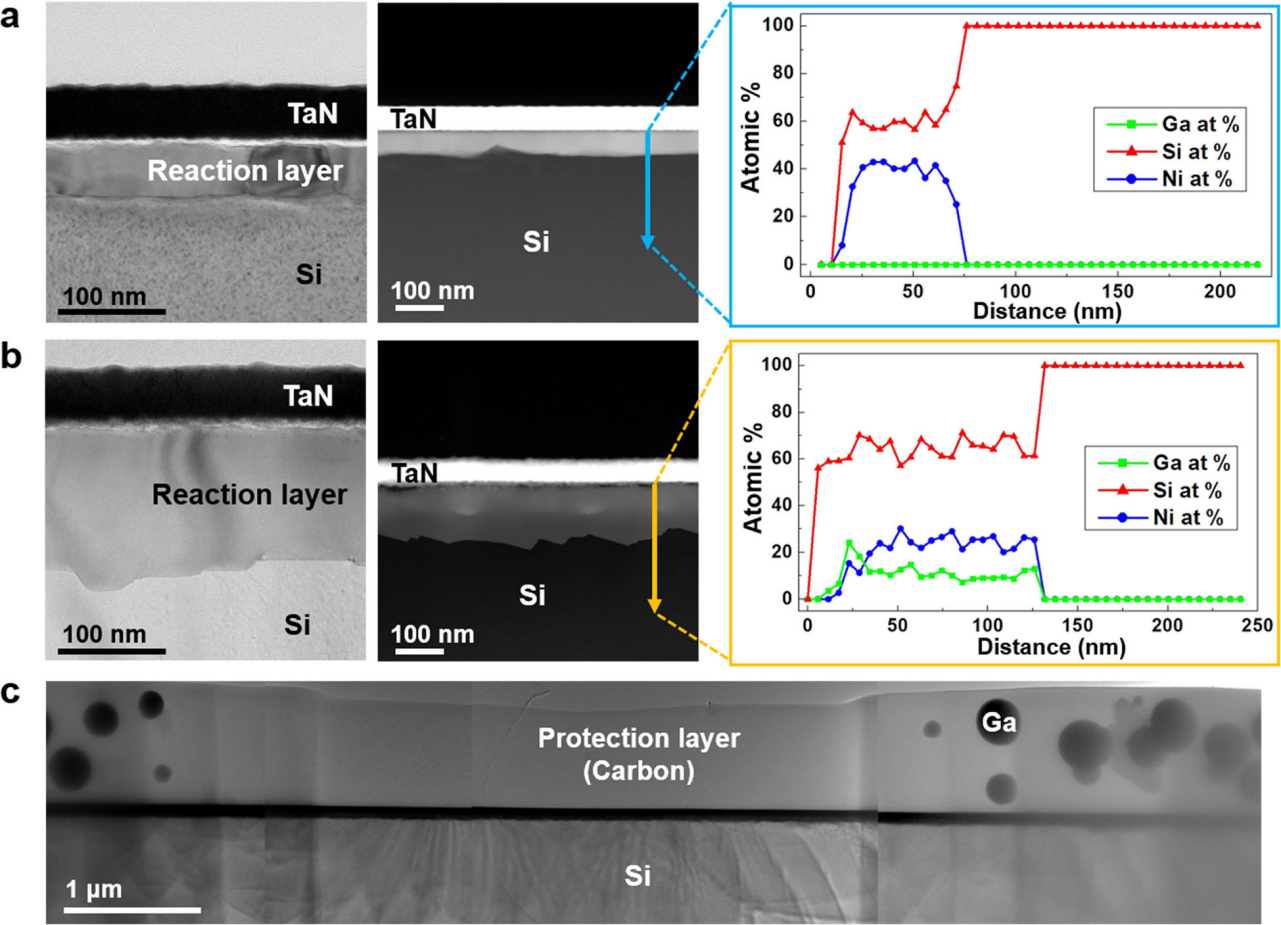
The TEM/high-angle annular dark-field (HAADF)-scanning transmission electron microscopy (STEM) images and EDS line profile of the heat-treated TaN/Ni/Si thin-foil specimen prepared via (**a**) mechanical polishing and ion milling, and (**b**) FIB milling/EXLO, (**c**) The low-magnification TEM image of the TaN/Er/Si thin-foil specimen prepared via FIB milling/EXLO

Initially, the silicide reactions after the thermal treatment of the Si-based binary thin-foil specimens that were fabricated by two different methods were compared. The TaN/Ni/Si thin-foil specimen that was prepared by mechanical polishing and ion milling showed a 50-nm-thick Ni-silicide reaction layer after the thermal treatment (Fig. 2(a)). The TaN/Ni/Si thin-foil specimen that was fabricated by FIB milling/EXLO showed a 114-nm-thick reaction layer after thermal treatment (Fig. 2(b)). The difference in the reactions that occurred in the two specimens was confirmed by the EDS line profile results. The presence of only Ni and Si was detected in the reaction layer of the former specimen (shown in Fig. 2(a)). However, the reaction layer of the latter specimen (shown in Fig. 2(b)) contained ~ 10 at.% Ga along with the elements that participated in the reaction. Although the two specimens were thermally treated under identical conditions, FIB milling of the latter thin-foil specimen resulted in the accumulation of the Ga residue that affected the silicide reaction. Furthermore, Ga^+^ ions were agglomerated in the form of a cluster on the protection layer of the heat-treated TaN/Er/Si thin-foil specimen (Si-based binary system) (Fig. 2c) that was fabricated using the same method as that for the specimen in Fig. 2(b). Therefore, Ar^+^ ion milling in the FIB system was utilized in this study for the necessary removal of the Ga residue.

**Fig. 3:**
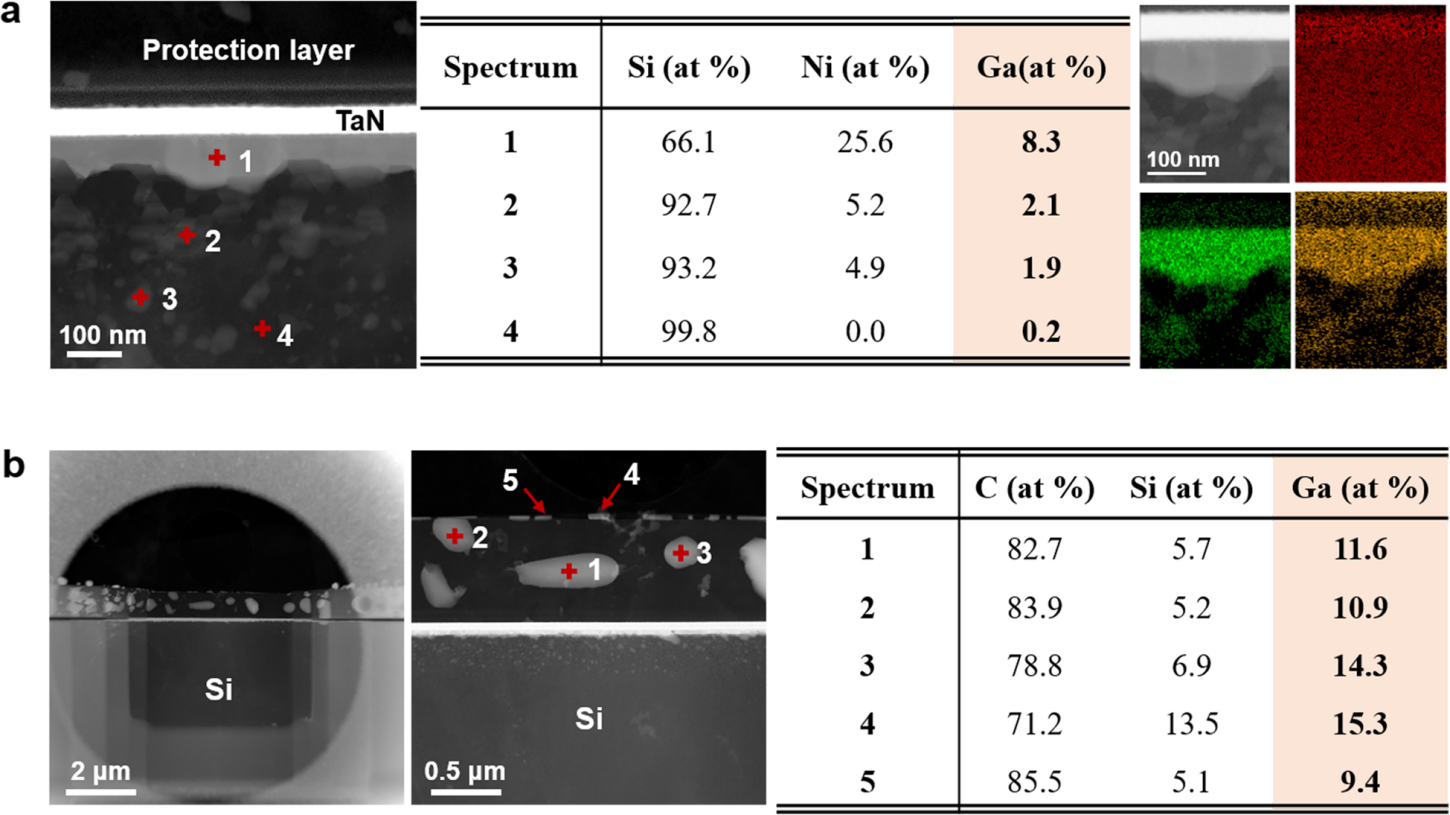
Results after the specimen in the trench is subjected to Ar^+^ ion milling. **a** The HAADF-STEM image and the results of the EDS analysis of the heat-treated graphene-encapsulated TaN/Ni/Si thin-foil specimen. The Si K series, Ni K series, and Ga L series EDS mapping is shown in red, green, and orange, respectively. **b** The HAADF-STEM image and chemical composition of the heat-treated graphene-encapsulated TaN/Er/Si thin-foil specimen

The thin-foil specimen was completely encapsulated using graphene to confirm the efficacy of the removal of Ga^+^ ions by Ar^+^ ion milling. Graphene is impermeable to all molecules except those of hydrogen gas; therefore, Ga^+^ ions could not escape from or enter the graphene-encapsulated specimen. Figure 3 shows the result of Ar^+^ ion milling of the thin-foil specimen inside the trench. Approximately 8.3 at.% Ga was detected in the Ni-silicide reaction of the graphene-encapsulated TaN/Ni/Si thin-foil specimen after thermal treatment (Fig. 3(a)). The EDS mapping also confirmed the presence of Ga in the region where Ni diffused onto the Si substrate. The Ga^+^ ions were primarily distributed in the protection layer, which was deposited during FIB milling, of the TaN/Er/Si thin-foil specimen after heat treatment (Fig. 3(b)). The detection of Si in the protection layer was attributed to the use of Fusion Thermal E-chips with a SiC substrate. The Ga residue formed irregular shapes in the graphene-encapsulated specimen, unlike the circular (2D) or spherical (3D) shape that were observed in the specimen shown in Fig. 2(c). The Ga residue also behaved like a viscous liquid due to its extremely low melting point of 29.8 °C and low surface free energy (Stevie et al. 2005). Therefore, the Ga^+^ ions moved together during the diffusion of the reacting substances due to the capillary forces of graphene; furthermore, the Ga residue formed irregular shapes due to negative volume expansion under pressure. Therefore, Ar^+^ ion milling was not effective for the removal of Ga^+^ ions from a specimen inside a deep trench. Most of Ga^+^ ions remained in such specimens and affected the reaction due to the redeposition of Ga^+^ ions or other materials on the specimen. Since Ar^+^ ion beam is not a focused beam, the Ar^+^ ions mill a considerably large area of the specimen. Therefore, the problem of the presence of the Ga residue will recur till the region of interest is located inside the trench, regardless of the rotation or tilting of the specimen stage. This phenomenon lowers the reliability of TEM analysis.

**Fig. 4:**
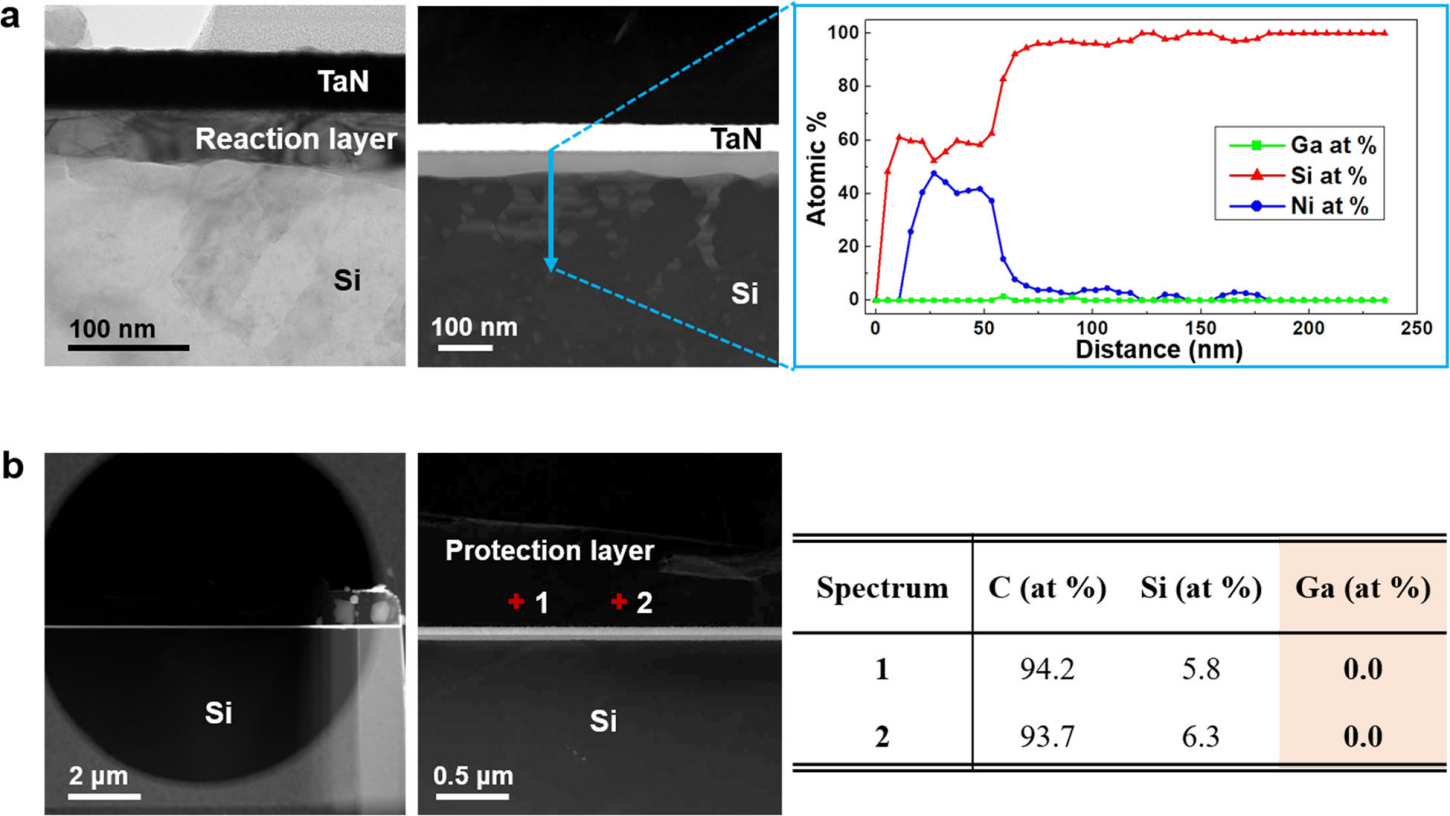
Result after the specimen attached to the FIB lift-out grid is subjected to Ar^+^ ion milling. **a** The TEM/HAADF-STEM images and EDS line profile of the heat-treated graphene-encapsulated TaN/Ni/Si thin-foil specimen. **b** The TEM/HAADF-STEM images and chemical composition of the heat-treated graphene-encapsulated TaN/Er/Si thin-foil specimen

To overcome aforementioned limitation, the specimen was attached to the FIB lift-out grid under specific conditions unlike those for the conventional FIB lift-out method, that are described in “Materials and Methods.” The specific conditions were required because the thin-foil specimen had to not only be fixed to the grid during futher milling and Ar^+^ ion milling but also be easily detached from the grid during the extraction by EXLO for the transfer onto the MEMS-based chip. The EDS analysis confirmed the absence of the Ga residue in the reaction layer of the TaN/Ni/Si specimen (Fig. 4(a)). Furthermore, the formed reaction layer was similar to that shown in Fig. 1(a). The concentration of Ga in the protection layer on the TaN/Er/Si specimen (Fig. 4(b)) was significantly low. Despite the presence of Ga in the thicker region of the specimen, the overall concentration of Ga remained low. It was concluded that the Ga^+^ ions were effectively removed by the method proposed in this study. The proposed method was convenient and allowed not only the completion of the entire milling process within the FIB system but also the thorough removal of Ga ions from both sides of the specimen.

## Conclusion

This study demonstrated the features induced by the accumulation of Ga^+^ ions in an MEMS-based chip specimen prepared by the conventional FIB milling/EXLO. The Ga^+^ ions influenced the binary system reaction and agglomerated in the protection layer. They also accumulated in the thin-foil specimen that was extracted from the trench by EXLO after Ar^+^ ion milling and affected the reactions during the in-situ experiment. To overcome this problem, we proposed a modified method of attaching the thin-foil specimen to an FIB lift-out grid by the IBID of carbon. Subsequently, this specimen was subjected to Ar^+^ ion milling and transferred to an MEMS-based chip by EXLO. The results of the EDS analysis indicated that most of the Ga residue in the specimen was removed by the proposed method.

## Data Availability

The datasets used and/or analyzed during the current study are available from the corresponding author on reasonable request.

## Abbreviations

TEM: Transmission electron microscopy
FIB: Focused ion beam
EXLO: ex situ lift-out
EDS: Energy dispersive X-ray spectroscopy
HAADF: High-angle annular dark-field
MEMS: Microelectromechanical
STEM: Scanning transmission electron microscopy

## References

[CR1] Campin MJ, Bonifacio CS, Nowakowski P, Fischione PE, Giannuzzi LA (2018). *Narrow-Beam Argon Ion Milling of Ex Situ Lift-out FIB Specimens Mounted on Various Carbon-Supported Grids*.

[CR2] Duchamp M, Xu Q, Dunin-borkowski RE (2014). Convenient preparation of high-quality specimens for annealing experiments in the transmission electron microscope. Microsc. Microanal..

[CR3] Giannuzzi LA, Drown JL, Brown SR, Irwin RB, Stevie FA (1997). Focused ion beam milling and micromanipulation lift out for site specific cross-section TEM specimen preparation. MRS Proc..

[CR4] Giannuzzi LA, Yu Z, Yin D, Harmer MP, Xu Q, Smith NS, Chan L, Hiller J, Hess D, Clark T (2015). Theory and new applications of ex situ lift out. Microsc. Microanal..

[CR5] Heringer LR, Chevacharoenkul S, Erwin DC (1996). *TEM Sample Preparation Using a Focused Ion Beam and a Probe Manipulator*.

[CR6] Ko DS, Park YM, Kim SD, Kim YW (2007). Effective removal of Ga residue from focused ion beam using a plasma cleaner. Ultramicroscopy.

[CR7] Mele L, Konings S, Dona P, Evertz F, Mitterbauer C, Faber P, Schampers R, Jinschek JR (2016). A MEMS-based heating holder for the direct imaging of simultaneous in-situ heating and biasing experiments in scanning/transmission electron microscopes. Microsc. Res. Tech..

[CR8] Pivak Y, Perez Garza HH, Zintler A, Molina-Luna L (2017). *FIB Lamella Sample Preparation on to MEMS Nano-Chips for In Situ TEM Applications*.

[CR9] Stevie FA, Giannuzzi LA, Prenitzer BI, Giannuzzi LA, Stevie FA (2005). The focused ion beam instrument. Introduction to Focused Ion Beams.

[CR10] Unoci KA, Mills MJ, Daehn GS (2010). Effect of gallium focused ion beam milling on preparation of aluminum thin foils. J. Microsc..

[CR11] Vijayan S, Jinschek JR, Kujawa S, Greiser J, Aindow M (2017). Focused ion beam preparation of specimens for micro-electro-mechanical system-based transmission electron microscopy heating experiments. Microsc. Microanal..

